# Shoulder Complex Dysfunction Through an Evolutionary Lens: The Need for Closed Kinetic Chain Loading in Upper Extremity Program Design

**DOI:** 10.3390/jfmk11020131

**Published:** 2026-03-24

**Authors:** David Luedeka, Keila Strick, Nickolas Roche, Caroline Williams

**Affiliations:** Fried Center for the Advancement of Potential, 5924 Fried Farm Rd, Crozet, VA 22932, USA; dluedeka@fcapotential.org (D.L.); kstrick@fcapotential.org (K.S.); nroche@fcapotential.org (N.R.)

**Keywords:** rotator cuff, evolutionary mismatch, evolutionary biomechanics, resistance training, shoulder stability

## Abstract

This review examines rotator cuff and shoulder complex dysfunction through an evolutionary framework and aims to translate these concepts into practical resistance training applications for strength and conditioning and rehabilitation professionals. Comparative anatomy and functional biomechanics of the human and non-human primate shoulder complexes are reviewed to illustrate how evolutionary pressures shaped an upper extremity system optimized for stability and force transmission under closed kinetic chain (CKC) loads. In contrast, many contemporary resistance training practices emphasize high-load, open kinetic chain (OKC) exercises that may impose elevated soft tissue strain and shear forces while potentially diminishing the engagement of the scapulothoracic and trunk stabilization mechanisms evolved to protect the shoulder complex. This proposed evolutionary mismatch may contribute to the high prevalence of shoulder dysfunction observed in the modern human population. Rotator cuff pathology arises through a combination of mechanisms, including, but not limited to, age-related tendon degradation, anatomical variations, mechanical overload factors, as well as systemic comorbidities. The contribution of habitual loading patterns to this multifactorial etiology has been considered in the literature, but this review advances a novel evolutionary mismatch hypothesis as one framework through which a primary biomechanical cause of overuse shoulder pathology may be examined. Applications of these concepts to exercise program design are presented. Specifically, training modifications consider moderately loaded CKC exercises performed at higher volumes with an emphasis on movement velocity and power generation. Incorporating moderate-load, high-volume, high-velocity CKC exercises may preserve rotator cuff integrity and optimize upper extremity function across the lifespan while potentially reducing the loading demands and associated mechanical stress that, under high-load or high-volume conditions, traditional OKC training models place on the shoulder and therefore, challenge the shoulder’s evolved structural tolerance.

## 1. Introduction

Shoulder pain is the third most common orthopedic problem for which individuals seek medical attention [1]. Injuries to the rotator cuff are one of the most common causes of shoulder pain, with almost 10% of individuals under 20 years of age and 62% of people 80 years of age or older experiencing rotator cuff dysfunction [2,3]. Standard treatment options for rotator cuff tears include non-surgical management, such as corticosteroid injections and physical therapy, or surgical intervention. Traditional physical therapy interventions for rotator cuff injury involve stretching to improve mobility and a variety of predominantly open kinetic chain (OKC) exercises (movements where the hands are free to move relative to a fixed body) aimed at increasing isolated strength in the rotator cuff and scapulothoracic musculature. Of those who opt for non-surgical care, long-term outcomes are questionable with possible continued progression of partial and full-thickness tears [2]. Surgical repair carries a risk of tear recurrence. While re-tear rates after rotator cuff surgery vary in the literature, a 2021 systematic review and meta-analysis found a re-tear rate of 15% within the first 3 months and 21% within 6 months of surgical intervention [3].

The failure of the rotator cuff’s ability to actively stabilize the glenohumeral joint results in an unstable fulcrum during humeral motion. Over time, this dysfunction can lead to rotator cuff arthropathy—a specific form of shoulder joint degeneration characterized by superior migration of the humeral head and progressive degenerative changes within the joint. This condition is commonly managed with a reverse total shoulder arthroplasty (RTSA). Since its approval in 2004, the use of RTSA has risen markedly. The number of RTSA performed in the United States nearly tripled between 2012 and 2017, increasing from 22,835 procedures to 62,075 [4].

The shoulder complex includes the structures of the glenohumeral, acromioclavicular, and sternoclavicular joints, the scapulothoracic gliding mechanism, and their associated periarticular structures [5]. Humans and non-human primates share a similar shoulder complex morphology, including anatomical risk factors that have been associated with subacromial impingement and rotator cuff tears [6]. Despite the high prevalence of rotator cuff dysfunction in humans, however, the prevalence of these pathologies is practically nonexistent in non-human primate research [7,8]. For example, assessment of 11 vervet monkeys ages 9 to 26 years old (representative of middle and later lifespan) found no sign of degenerative rotator cuff tears [9]. Rotator cuff degeneration is recognized as a multifactorial process, with contributing factors that include intrinsic age-related tendon changes, reduced vascularity, repetitive overhead or high-load demands from occupational or recreational activity, metabolic and systemic influences, and genetic predisposition [10]. The pronounced disparity in pathology rates between humans and non-human primates, despite broadly shared shoulder morphology and similar anatomical risk factors, raises the question of whether biomechanical factors specific to modern human activity patterns may represent an important, unexplored contributor to this burden.

The authors propose that one potential strong contributing factor may be an evolutionary mismatch: the modern human shoulder complex retains morphological features optimized for closed kinetic chain (CKC) loading patterns where the hands are fixed and the body moves relative to them (e.g., quadrupedal weight-bearing and branch ambulation). The evolved role of the human upper extremity during bipedal gait, along with modern daily activity and training practices place predominantly repetitive—and often heavy—OKC loads on the shoulder. (e.g., bench press and overhead arm motion). The way modern humans use their upper extremities in today’s culture may create loading patterns that are hypothesized to be less matched to our evolved shoulder structure, and may therefore impose loading demands that the joint’s evolved morphology was not primarily optimized to accommodate. We hypothesize that, over time, this theorized mismatch may cumulatively stress the rotator cuff in ways that could contribute to the high prevalence of overuse orthopedic injury observed in humans, though direct causal evidence remains to be established. It should be noted that resistance training, including OKC exercises, can be performed safely and can offer significant musculoskeletal benefits when appropriately programmed. The evolutionary mismatch hypothesis, instead, proposes a potential risk that appears specific to humans in the case of high-load conditions, suboptimal technique, or overuse. While the relationship between anatomical form, function, and rotator cuff pathology of the upper extremity in human and non-human primates has been explored in the literature, these relationships have not been formally considered for clinical application [8].

Questionable treatment outcomes and rising surgical intervention rates underscore the need to better understand the root cause of rotator cuff pathology in humans, as well as develop effective injury prevention strategies. This narrative review aims to: (1) examine the comparative anatomy and functional biomechanics of the primate shoulder complex, demonstrating how evolutionary forces shaped structures optimized for CKC loading patterns; (2) examine whether certain modern resistance training practices, particularly high-load OKC exercises such as the bench press and lat pull-down, may generate excessive soft tissue strain and shear forces while decreasing engagement of the integrated scapulothoracic and core stabilization mechanisms that evolved to protect the shoulder complex. Based on these findings, the authors suggest theoretically informed training modifications aligned with the evolutionary mismatch hypothesis, emphasizing moderate-load CKC exercises. They also provide specific programming recommendations intended to better match the structural and functional constraints of the shoulder girdle, with the goal of preserving rotator cuff integrity and optimizing upper extremity function across the lifespan.

## 2. Evolution of the Human Shoulder Complex

Over the course of approximately 55–80 million years, evolutionary pressures shaped the anatomy of all primates. When *Homo sapiens* diverged from other hominins roughly six million years ago, substantial structural adaptations occurred in the lower extremities and spine to support the emergence of habitual bipedalism. These changes facilitated more efficient locomotion and are thought to have evolutionary advantages related to increased foraging range and improved energy conservation [11].

Primate shoulder morphology varies widely in response to differing functional demands and habitual modes of locomotion. Variations in the primate shoulder complex reflect the need to accommodate distinct combinations of tensile, compressive, and shear forces. For example, the shoulder complex of a baboon differs markedly from that of an orangutan: baboons primarily engage in terrestrial quadrupedalism, whereas orangutans move through their environment by swinging beneath branches. The following sections summarize key shoulder morphological features associated with quadrupedal and suspensory locomotor behaviors, with particular attention to adaptations that attenuate the external forces encountered during these activities.

Quadrupedalism is the most common form of locomotion among primates and involves walking or running on all four limbs. This locomotor pattern can be broadly categorized as terrestrial or arboreal. Terrestrial quadrupedalism is characterized by movement over ground and is exemplified by baboons [12]. Chimpanzees and gorillas represent a specialized subtype of terrestrial quadrupedalism known as knuckle walking, during which approximately 20% of body weight is transmitted through the forelimbs. During terrestrial quadrupedism, the upper extremities are primarily subjected to compressive and shear forces, and shoulder morphology reflects adaptations to enhance joint stability and accommodate these loads. For example, terrestrial quadrupeds feature a flattened superior humeral head to improve contact with the glenoid of the scapula [13,14], a more proportionate glenoid and humeral head size resulting in a relatively larger contact area between glenohumeral joint surfaces that spreads joint contact force over greater area and decreases focal stress within the joint [15]. Humeral tubercles positioned superior to the humeral head yield increased rotator cuff mechanical advantage through an increased moment arm and enhanced torque production about the glenohumeral joint [16,17]. Additional morphological features associated with quadrupedal primates are listed in Table 1.

Arboreal quadrupedalism involves movement along tree branches and is commonly used by primates such as squirrel monkeys. In this mode of locomotion, the upper extremities experience compressive and shear forces like those seen in terrestrial quadrupedalism, but are also exposed to tensile forces associated with branch grasping and balance [12]. As a result, the shoulder complexes of arboreal quadrupeds must balance stability with sufficient mobility to allow effective navigation through the arboreal environment. While the shape of the humeral head, glenoid, scapula and thorax are similar to terrestrial quadrupedal primates, arboreal quadrupeds feature a slightly more globular humeral head and humeral tubercles that are more inferior than terrestrial quadrupeds, adaptations that allow for increased mobility [24,25].

Suspensory brachiation is a mode of locomotion in which primates grasp and swing beneath branches using their forelimbs [12]. Gibbons, spider monkeys, and orangutans are examples of primates that habitually engage in suspensory locomotion. During brachiation, the upper extremities are exposed primarily to CKC tensile forces, and shoulder morphology is adapted to optimize mobility and range of motion in overhead positions. These adaptations include a wider thorax and scapula oriented in the frontal plane [26]. The humeral head is globular and articulates with a smaller, superior-facing glenoid [27,28]. The result is that only 25–30% of the humerus is in contact with the glenoid [27,29]. The tubercles of the humerus are positioned inferior to the humeral head, which decreases the mechanical advantage of the rotator cuff musculature while allowing for increased range of motion. The infraspinatus has a more superior attachment on the greater tubercle, allowing it to act as a glenohumeral stabilizer against distraction forces experienced during hanging [16,17,18,22]. Additional morphological features associated with suspensory-brachiating primates are listed in Table 2.

Humans are members of the hominid family, which also includes chimpanzees, gorillas, bonobos, orangutans, and extinct human and ape ancestors. Within this family, there is substantial diversity in shoulder complex morphology and habitual locomotor behavior. Although several non-human primates are capable of bipedal gait, humans are uniquely efficient bipedal ambulators and are the only primates for whom bipedalism serves as the primary mode of locomotion. In contrast to the significant differences observed between humans and non-human primates in the pelvic and lower-extremity anatomy, the upper extremities exhibit relatively minimal structural differences. Human transition to habitual bipedalism substantially reduced the CKC demands associated with quadrupedal and suspensory locomotion and shifted upper-extremity function toward predominantly OKC activities performed at relatively low loads, such as holding, grasping, and manipulating objects [8]. Existing shoulder morphology was largely sufficient to accommodate these functional demands [21,27]. As a result, the human shoulder complex most closely resembles that of suspensory-brachiating primates and is characterized by a high degree of mobility. Like their suspensory-brachiating ancestors, humans possess a dorsally positioned scapula in the frontal plane. It rests on a broad wider thorax. The humeral head is large and globular with increased medial–lateral dimensions, allowing for greater external rotation, which is needed for greater tubercle clearance with shoulder elevation [23]. Humans also demonstrate minimal glenohumeral contact surface area and similar attachment positions of the supraspinatus, infraspinatus, and teres minor to suspensory-brachiating primates [33,34].

**Figure 1:**
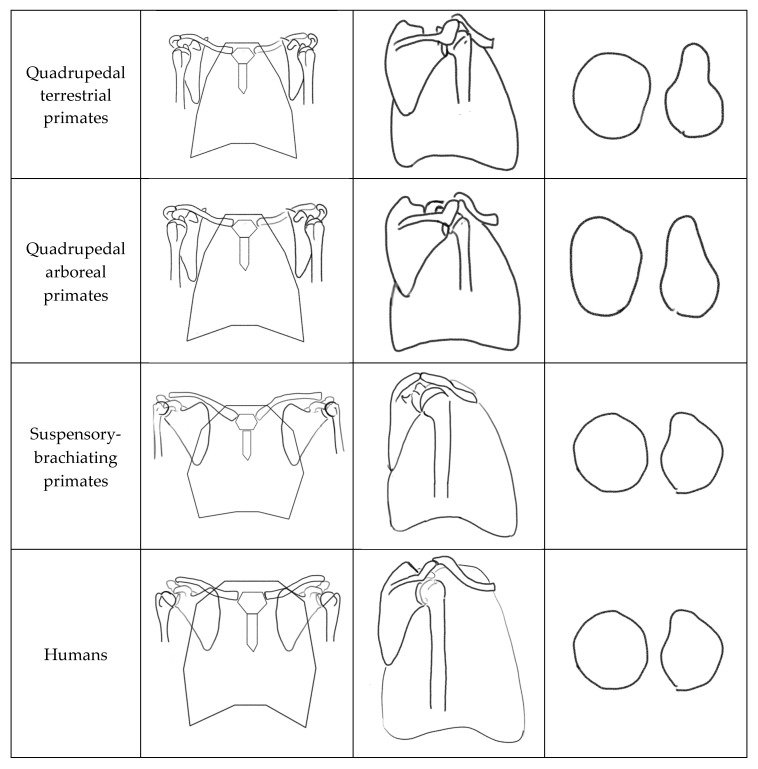
Morphological features of the primate shoulder complex categorized by primary ambulation type. The figure illustrates distinctive structural characteristics associated with locomotor patterns across primate species. This is an original adaptation [19].

Although humans retain several morphological features of suspensory-brachiating primates, important distinctions remain. In suspensory-brachiating primates, the scapula is oriented with the glenoid facing more superiorly, whereas in humans it faces laterally [19,30,32]. This results in a higher functional center of abduction in suspensory-brachiating primates, facilitating safer overhead loading and reducing the risk of supraspinatus impingement. The more inferior human shoulder abduction fulcrum position may suggest reduced selective pressure for extreme overhead loading capacity, though this inference is based on morphological comparison rather than direct functional measurement [21]. Additionally, human shoulders exhibit decreased greater tubercle volume at the insertion sites of the supraspinatus, infraspinatus, and teres minor, and reduced supraspinatus muscle mass compared to other hominids [24,35]. While direct comparative biomechanical data remain limited, these anatomical differences are consistent with the theoretical inference that rotator cuff loading demands across species, given that greater muscle mass and larger attachment areas suggest higher functional loading requirements. These morphological features have been theorized to reflect evolutionary changes associated with the transition to bipedalism, which may have reduced the upper extremity’s dependence on rotator cuff-mediated glenohumeral stabilization [8,24]. Main morphological distinctions between primate shoulder complexes based on primary modes of locomotion are illustrated in Figure 1.

## 3. Closed Kinetic Chain Muscle Function and Modern Strength Training Analysis of the Shoulder Complex

Although the modern human upper extremity is predominantly used in OKC tasks, the primate shoulder evolved to support locomotion under CKC conditions, including quadrupedal and suspensory behaviors. In climbing, brachiating, and scrambling primates, the dynamic stability of the shoulder complex relative to the axial skeleton is essential for effective force transmission and survival.

This functional importance is reflected in the muscular redundancy found in the primate shoulder girdle. The trapezius muscle spans from the occiput to the thoracic spine and is functionally subdivided into three regions. Each part functions in a comparable manner to muscles deep to it; for instance, the rhomboids and the middle trap both retract the scapula. Notably, these deeper structures are innervated by branches of the brachial plexus, while the trapezius is unique in its dual innervation by the spinal accessory nerve and cervical spinal nerves. In other words, injury to a single component is less likely to result in failure of the overall movement system.

To evaluate whether contemporary training practices align with inherited shoulder structure, it is necessary to distinguish between OKC and CKC movement patterns. A key difference lies in the integration of proximal musculature. CKC upper-extremity exercises require coordinated activation of the trunk and scapulothoracic muscles, facilitating force transfer across segments, and enhancing glenohumeral joint stability. Proximal stability combined with agonist–antagonist co-contraction limits excessive accessory joint motion, supports a more stable axis of rotation, and optimizes connective tissue length–tension relationships, thereby improving shoulder joint biomechanics.

Modern culture forces the shoulder joint complex to function in ways that deviate from the CKC forces that shaped its evolved structure. Our upper extremity structure is often challenged by repetitive, overhead, and heavily loaded OKC motions that create increased stress on soft tissue structures during occupational activities. This stress is also prevalent in the current strength and conditioning environment. In the 1950s, powerlifting was officially sanctioned by the Amateur Athletic Union (AAU), with the deadlift, squat, and bench press designated as the core competitive lifts. By the 1980s, powerlifting had gained mainstream popularity, and these lifts—particularly the barbell bench press—became a foundational tool for building upper body strength and enhancing athletic performance. Today, the bench press is widely regarded as the standard for upper-body strength development. However, injury rates during common power lifting activities find the greatest prevalence of injury reports occur during the bench press [36].

Common injuries sustained during the bench press include distal clavicular osteolysis, pectoralis major rupture, rotator cuff tendon injuries, biceps tendon injuries, and labral tears [37].

The following section compares two pairs of exercises that represent common OKC and CKC shoulder-loading strategies: pushing movements (bench press versus push-up) and pulling movements (lat pull-down versus pull-up).

### 3.1. Review of the Bench Press Exercise

For most users, the goal of the bench press exercise is to create hypertrophy and improve the performance of the chest and triceps musculature. During the bench press, maximal pectoralis major muscle activation occurs with a wide hand placement (defined as 2 biacromial widths) and shoulder abduction angles of 90 degrees [33,37]. Highest 1 rep max performances are typically achieved with this position as well [38]. Therefore, it would be logical that one would perform a bench press with a wider grip to challenge the chest musculature and increase strength. The following section examines the potential biomechanical stressors experienced during the bench press.

Human shoulder morphology most closely mimics arboreal primates, which feature a very globular humeral head and a smaller glenoid fossa compared to terrestrial primates [27,29]. This results in only 25% to 30% of glenohumeral joint surfaces in contact at any given time [27,29]. There is a component of shoulder joint stability in the shoulder that occurs from joint surface compression—when the humeral head is compressed into the glenoid, resistance to shear forces and accessory motion increases [39]. In addition to increased glenohumeral stability, light to moderate compressive loads promote healthy articular cartilage [40]. However, excessive compressive loads, especially in combination with increased shear force, can be damaging to articular cartilage and increase the risk for articular cartilage pathology [40]. Noteboom et al. evaluated glenohumeral joint compression forces during the bench press using a 35 lb barbell and varying hand placements. Their findings demonstrated that internal joint compression increased with increasing grip width, with the highest compressive forces observed at a grip width of 2.0 times the biacromial distance [37]. These results suggest that hand placement may substantially influence internal joint loading during pressing movements. Further investigation is warranted to better characterize internal joint pressures during comparable closed-kinetic-chain exercises, as well as to evaluate glenohumeral joint pressures during the bench press under heavier loads that more closely reflect contemporary resistance training practices. Appendix A presents a theoretical estimation of internal glenohumeral joint pressures during a 200 lb bench press and examines their potential relationship to reported thresholds of maximal articular cartilage pressure tolerance.

Mobility and positioning of the thoracic cage, scapulothoracic joint, and glenohumeral joints are interdependent, with decreased scapulothoracic mobility correlating with increased glenohumeral motion [41]. When lying on a bench, the scapulohumeral rhythm is disrupted. Not just from the physical restraint of the bench itself, but because the scapulothoracic and core muscles are not as engaged due to the bench providing external support to the center of mass. Studies demonstrate increased electromyography (EMG) muscle activation in the core and scapulothoracic muscles when comparing closed versus open chain exercises [34]. The engagement of core and scapulothoracic muscles helps create a coordinated and effective transfer of force across segments, enhancing glenohumeral joint stability and decreasing accessory joint motion [42,43]. EMG signals are commonly used to evaluate neuromuscular activation by measuring the electrical signals associated with motor unit recruitment within a muscle; higher EMG amplitudes generally reflect greater motor unit recruitment and increased muscular activation. In a study by Noteboom et al., rotator cuff EMG activity was examined during the bench press performed with light loads (35 lbs) in healthy, experienced athletes using a standardized setup across grip widths to control for individual variability in adaptive response [37]. The authors reported that a wide-grip bench press produced a 22% increase in overall rotator cuff EMG activity compared with a narrow grip, which was interpreted as increased engagement of the active glenohumeral stabilizers to counter shear forces at the shoulder joint. Notably, supraspinatus activity increased by 41% and accounted for approximately 37% of total rotator cuff activation during the wide-grip condition [37]. Although surface EMG does not directly reflect tissue strain or mechanical load [44,45,46], it is widely used to evaluate neuromuscular coordination and identify altered muscle activation patterns or compensatory recruitment strategies during functional movement tasks [47,48,49]. From an evolutionary perspective, the supraspinatus of *Homo sapiens* is the least robust among primates [24], suggesting that exercises requiring substantial supraspinatus-mediated stabilization—such as the wide-grip bench press—may warrant careful consideration.

Performing the bench press with a narrower grip and a retracted scapula, as recommended by the National Strength and Conditioning Association, can reduce glenohumeral joint compression and shear force [37]. However, this does not account for disruptions to shoulder complex arthrokinematics. Calculating a relative injury risk due to excess compression and shear forces experienced during the wide-grip bench press is an area of research that requires more investigation. However, training to increase pectoralis major hypertrophy and strength while optimizing shoulder biomechanics justifies exploration of alternative training methods.

### 3.2. Review of the Push-Up Exercise

**Figure 2:**
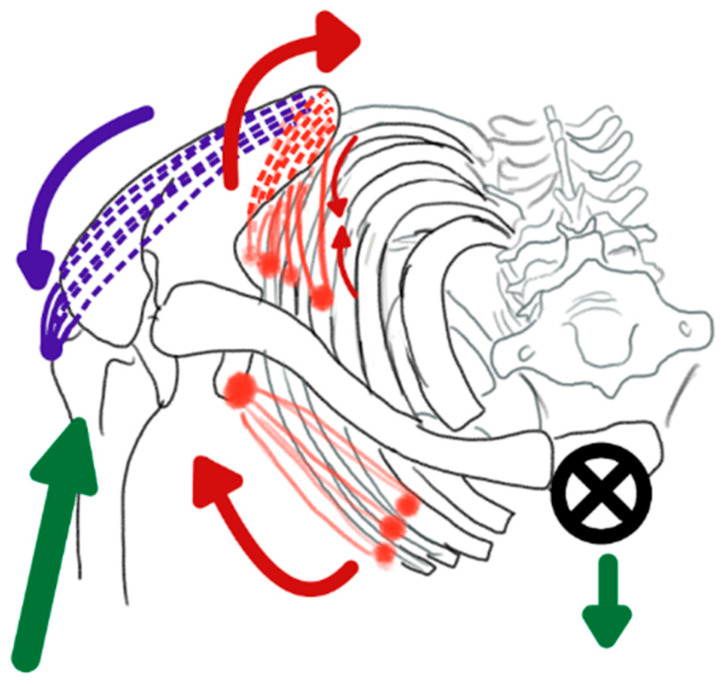
Internal and external forces present during push-ups. External forces are depicted by green arrows and internal muscular forces by red and purple arrows. The ground reaction force (green arrow directed through the humerus) produces a moment that tends to lift the lateral border of the scapula away from the thorax. The pectoralis minor (red, anterior) stabilizes this force by drawing the scapula dorsally, approximating the lateral scapular border against the thorax. The gravitational force (green arrow directed downward through the center of mass) pulls the thorax toward the ground. The serratus anterior (red, posterior) stabilizes this load by drawing the thorax toward the medial border of the scapula, creating an external rotation moment at the scapula. The infraspinatus (purple, posterior) counteracts this external rotation by generating an internal rotation force to maintain scapular stability.

The push-up and the bench press both cause compression through the glenohumeral joint, either through ground reaction forces or the weight of a barbell. The push-up and bench press also demonstrate similar activation patterns in the primary muscles targeted during both movements [50,51]. If standardized for load and training volume, the push-up and bench press produce comparable gains in strength and muscular hypertrophy [52]. However, there are significant differences that suggest push-ups may be preferred for optimal shoulder health. When the body’s center of mass is exposed to gravity without external support, as in a push-up, engagement of core and scapulothoracic musculature increases. There seems to be a heightened neurological response in the scapulothoracic musculature when the core is engaged due to its exposure to gravity [53]. This activation helps create a coordinated activation of scapular and trunk musculature, which allows for effective transfer of force across joined segments and maintains stable contact between the glenoid and the humerus [42]. These internal and external forces are illustrated in Figure 2.

In contrast to the bench press, maximal pectoralis major activation is achieved with a close hand placement, which also places comparatively less demand on the supraspinatus muscle [37,51]. Another potential benefit is that pectoralis muscle activation is greatest during narrow-grip push-ups [51]. This may be associated with the preference of our arboreal ancestors to stabilize the shoulder joint through the adductors of the humerus due to the dorsal placement of the scapula on the thorax and the adductor force required to grip a branch [19]. While there are no known studies that investigate internal joint pressures during push-up variations, Noteboom et al. found glenohumeral joint contact forces were minimized during a narrow grip bench press [37]. While activation of rotator cuff musculature is still required during a narrow grip push-up, there is less reliance on the supraspinatus when compared to a bench press. Push-ups also appear to require relatively lower rotator-cuff mediated demand from the supraspinatus compared to the bench press—in fact, the infraspinatus demonstrates almost three times more activity than the supraspinatus during a push-up [54]. Normalized CKC arthrokinematics may play a role in increased infraspinatus activity. It is theorized by these writers that gravitational forces acting on the body’s center of mass require serratus anterior engagement, which draws the thorax toward the scapula and creates an external rotation moment at the scapula during the push-up. During open chain movement, the infraspinatus is understood to be a humeral external rotator. However, in a closed chain, its attachment creates an internal rotation torque at the scapula, which helps maintain stability and normal biomechanics at the shoulder joint during the narrow grip push-up.

### 3.3. Review of the Pull-Up

Although both pull-ups and lat pull-downs target the latissimus dorsi and elbow flexors, their loading contexts differ substantially. The pull-up more closely replicates the suspended, CKC conditions under which human shoulder morphology evolved. In this context, distraction forces act through the shoulder and trunk, necessitating greater activation of the core and scapular stabilizers compared to the lat pull-down. Consequently, pull-ups elicit greater trunk and core muscle activation than comparable OKC exercises [34,55]. Despite higher external distraction forces, McGill and colleagues found that pull-ups and chin-ups demonstrated greater spinal compression than comparable exercises where feet were supported. Increased spinal compression may be related to the increased activation of the rectus abdominis and lats required during these exercises [55]. This proximal stability provides a foundation for efficient force transmission through the shoulder complex [42].

During pull-ups, proximal stability precedes distal mobility, optimizing movement coordination. The latissimus dorsi, with its extensive attachments to the vertebrae, thoracolumbar fascia, pelvis, and ribs, functions as both a prime mover and a stabilizer across multiple segments. Kinematic analyses of the pull-up find thoracic elevation precedes humeral depression, reflecting a proximal-to-distal movement pattern. Latissimus dorsi contraction compresses the humeral head into the glenoid while producing scapular depression and downward rotation [56]. In response, the upper and lower trapezius generate upward rotation to counterbalance this force, contributing to dynamic scapular stability.

**Figure 3:**
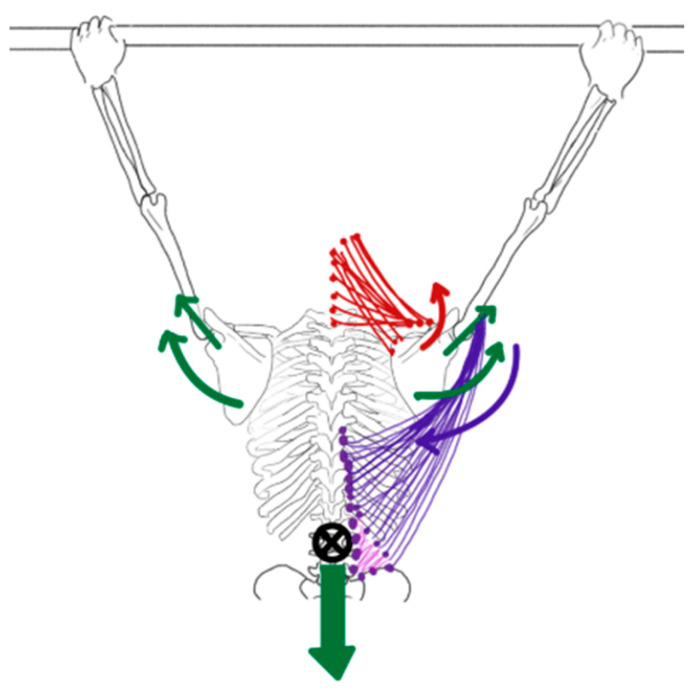
Internal and external forces present during pull-up. External forces are depicted by green arrows and internal muscular forces by red and purple arrows. The gravitational force (green arrow directed downward through the center of mass) generates distraction and tensile loading at the glenohumeral joint and across the scapulothoracic interface. The latissimus dorsi (purple, lateral) draws the thorax toward the humerus, stabilizing the humeral head within the glenoid while producing scapular depression and downward rotation through contact forces. The upper trapezius and levator scapulae (red, superior) counteract this downward rotation moment by generating an opposing upward rotation force at the scapula to maintain scapular stability.

Gravity further challenges scapulothoracic stability during pull-ups by attempting to position the center of mass directly beneath the fixed hand position. This force tends to dissociate the scapula from the thorax, prompting increased serratus anterior activation to stabilize the scapula against the rib cage [55,56]. Concurrently, the rhomboids and middle trapezius contract eccentrically to control scapular elevation [56]. These coordinated co-contraction patterns likely reduce rotator cuff impingement by maintaining optimal humeral head positioning within the glenoid. These internal and external forces are illustrated in Figure 3.

### 3.4. Review of the Lat Pull-Down

In contrast, the lat pull-down is typically performed seated with external torso stabilization and an OKC movement pattern. External support reduces the need for core engagement and diminishes activation of key scapular stabilizers [34,55]. Reduced recruitment of the erector spinae, deep spinal stabilizers, and abdominal musculature compromises proximal stability, potentially altering shoulder joint mechanics and increasing stress on the rotator cuff.

Current research has not directly examined these mechanisms. However, the authors speculate that closed-kinetic-chain (CKC) pull-ups and chin-ups may be more beneficial for individuals with shoulder pain than open-kinetic-chain (OKC) lat pulldowns. This hypothesis is based on differences in how the shoulder is loaded during the overhead phase of each exercise. During the OKC lat pulldown, the arm moves overhead with relatively passive control as the weight stack rises. This may reduce the need for eccentric activation of the latissimus dorsi while the arm is elevating. With less active muscular control, the humeral head may migrate superiorly, potentially increasing stress within the subacromial space. In contrast, CKC exercises such as pull-ups and chin-ups require the body to move relative to fixed hands. This likely necessitates greater continuous activation of the latissimus dorsi to control humeral position as the arm moves into elevation. The authors speculate that this increased activation may improve humeral stabilization and reduce superior humeral head migration. This reasoning is also informed by comparative anatomy. In arboreal primates, the latissimus dorsi functions as a powerful stabilizer of the humerus during climbing and overhead locomotion. Accordingly, the authors propose that strong latissimus activation may similarly help stabilize the humerus during overhead movement in humans, potentially supporting shoulder mechanics during CKC pulling tasks.

## 4. Training Metrics to Support Lifelong Shoulder Complex Health

The aging process is highly heterogeneous, shaped by complex interactions among genetic, environmental, behavioral, and demographic factors, and no single, unifying theory of biological aging currently exists [57,58]. Yet, predictable age-related changes occur across all physiological systems, impacting muscle, bone, cartilage, and nervous system tissues. These changes can reduce overall physiological reserve, affect strength, power, range of motion, and functional capacity, and increase vulnerability to non-communicable chronic diseases [59,60]. For example, although osteoarthritis is not an inevitable consequence of aging, the prevalence of arthritis in the United States increases sharply with age, from 3.6% in adults aged 18–34 to 53.9% in adults 75 years and older [61].

As the average global life expectancy continues to increase [62], prioritizing musculoskeletal and joint health across the lifespan is essential. Exercise is widely recognized as the most effective intervention for enhancing physical, psychological, and cognitive health in older adults, with benefits observed across all ages and stages of initiation [63,64]. While traditional resistance training approaches at heavy loads are effective to maximize strength gains [65], they may not be optimal to protect joint integrity. This concern is especially relevant for the shoulder complex, as its evolutionary development under primarily CKC demands may limit its tolerance for repetitive heavy OKC loading. The following section presents an evolutionary-informed training framework that accounts for the morphological and functional realities of the human shoulder girdle. The authors posit that progressive resistance exercise programs performed under moderately loaded CKC forces, at a high training volume, and with an emphasis on power generation through overspeed training, will address the evolutionary mismatch of the upper extremity while supporting lifelong physical health and function.

### 4.1. Power Generation and Muscle Composition

Power is defined as the ability to move a load over a given distance within a specific time. Power production is dependent on contributions from the neuromuscular system and both the central and peripheral nervous systems. Power generation is critical for activities of daily living and is a stronger predictor of physical function, fall risk, and frailty than either muscle mass or strength [66]. In older adults, power declines more rapidly than strength and muscle mass [67,68,69]. On average, about 67% of chimpanzees’ muscle fibers are characterized as Type II (fast twitch), compared to roughly 50% in humans. This difference may help explain why chimpanzees can generate about 1.35 times more power and dynamic force than a human muscle of comparable mass [70]. Maintaining higher overall muscle mass and fat reserves requires greater energy expenditure, which would have been disadvantageous in our evolutionary past at a time when calories were scarce. Fast-twitch fibers allow for short bursts of force production without the metabolic cost of significantly larger muscles. In contrast, human evolution appears to have favored a muscle fiber composition optimized for endurance rather than power. With the adoption of bipedal gait, ambulation became more energy-efficient, enabling humans to travel longer distances [71]. It is important to note that cross-species comparisons should be interpreted with caution, given the translational limitations of applying comparative morphology to modern human training.

Type I (slow-twitch) fiber contraction speed is a significant contributing factor in power and rate of force development (RFD), among other factors like motor unit recruitment strategy, rate coding, musculotendinous stiffness, muscle activation, and muscle size and architecture [72]. According to Henneman’s size principle, motor units are recruited from smallest to largest based on force demands, meaning type I fibers are activated first during low-force tasks, with progressively larger type II fibers recruited as force requirements increase [73]. Although humans have retained many structural features of the primate shoulder complex, we did not retain their higher fast-twitch fiber volume, which limits our capacity for rapid force production [70]. Importantly, resistance training aimed at power can promote adaptations toward faster fiber characteristics and help maintain fast-twitch function, which is particularly critical with aging, as these fibers support power, balance, and rapid reactions. Many training protocols demonstrate improvements in power and RFD through strength training, yet most do not adequately consider long-term joint health [74]. Consequently, we propose that high-speed, moderate-load training is a critical component for preserving fast-twitch fibers, optimizing power, and maintaining functional performance, and that training programs must intentionally address this physiological limitation [74,75].

Optimizing power requires balancing force production with movement speed, as moving heavier loads too slowly diminishes power output. Evidence suggests that the optimal load for power development is approximately 40–60% of an individual’s 1-repetition maximum (1 RM) [76,77]. Increases in maximal strength do not necessarily result in faster movements [78,79]. This highlights the need to specifically target movement speed to improve power.

Muscle fibers adapt to training demands: studies found heavy, slow resistance training over 6–16 weeks tends to shift fast-twitch fibers toward more endurance-oriented types, whereas fast, explosive training helps preserve and enhance fast-twitch characteristics [74,75]. For example, research on athletes performing 6 ssprint intervals at high volumes (90 intervals per session) led to “a nearly complete slow-to-fast fiber transformation on the mRNA level” with significant performance improvements in both power and endurance performance [80]. However, it is important to note that fiber type transitions exhibit considerable inter-individual variability influenced by genetics, age, training history, and program design [74].

Research further supports the benefits of power training for improving strength, power generation, muscle mass, and functional capacity [81]. In fact, high-velocity training has been shown to produce greater improvements in functional outcomes than traditional resistance training, regardless of load [66].

Overspeed training—where body weight is reduced to allow movements at otherwise unattainable velocities—also shows promise for enhancing power and speed. Although research in older, non-athletic populations remains limited, ref. [82] findings to date are encouraging. For example, a randomized controlled trial of 22 healthy women aged 75–85 demonstrated a 13% increase in walking speed and a 67% increase in mechanical power after 12 weeks of treadmill overspeed training with body-weight support [83]. Similarly, high-speed resistance training (HSRT) at moderate loads (10–50% 1 RM) has been shown to improve both physical and cognitive function in older adults [84,85].

### 4.2. Load and Volume

Training strategies that emphasize maximal speed at low to moderate loads may not only improve power production and facilitate long-term joint health, but may also improve lean tissue metrics. Training to preserve function requires an optimal load, most often expressed as a percentage of an individual’s 1 RM. Ideal load stimulus challenges the musculoskeletal system enough to elicit tissue adaptation but does not exceed the connective tissues’ injury threshold [86]. Research suggests that performance enhancement in strength, hypertrophy, and power outcomes can be achieved at submaximal loads if muscles are trained at or near volitional failure [87,88,89]. In addition, one of the referenced studies also found that levels of muscle activation (estimated via amplitude of glycogen depletion) and hypertrophic response (via anabolic protein signaling) for both Type I and Type II muscle fibers did not differ between participants who trained at 30% versus 80% of their 1 RM as long as the exercise was performed to failure [88].

Volume plays a key role in muscle tissue adaptation. There is a balance between load selection and training volume. As training load decreases, volume (defined as the weekly number of repetitions and sets of training per muscle group) must increase to stimulate muscle adaptation. Current evidence supports a dose–response relationship between muscle adaptation and weekly training volume [87,90]. One study found maximal increases in muscle hypertrophy when training volume met or exceeded 10 weekly sets per muscle group [90]. Another study identified 12–20 weekly sets per muscle group as an optimal range for muscle hypertrophy in resistance-trained individuals [87]. In practice, this implies matching moderate loads performed with intent for velocity to a sufficient weekly set volume to achieve proximity to failure while respecting joint tolerances.

Consistent with this literature, moderate-load, high-velocity training within CKC patterns is emphasized to support force production, movement speed, and tissue tolerances. However, there are currently no longitudinal trials directly comparing joint health outcomes across these training paradigms (e.g., CKC vs. OKC). Accordingly, this training approach is offered as a programmatic framework informed by current evidence, shoulder girdle biomechanics, and clinical reasoning, recognizing that direct longitudinal comparisons across paradigms remain limited.

## 5. Limitations

Several factors limit the strength, reproducibility, and generalizability of the findings and conclusions presented in this review. As a narrative review, this work does not employ a systematic search strategy or formal risk-of-bias assessment, which may introduce selection bias and limit complete representation of the existing literature. Second, the evolutionary mismatch theory of the modern human shoulder—and its application to the adaptation of training and rehabilitation techniques—remains a relatively novel framework. Many aspects of this approach diverge from conventional strength training practices, and the current empirical support is less robust than that available for traditional training methods. Relatedly, some proposed biomechanical mechanisms are supported by emerging evidence that remains limited in clinical research.

Although these factors limit the ability of the present review to infer causation or predict long-term outcomes, this work aims to introduce an innovative framework for upper extremity training and rehabilitation and to provide a conceptual foundation for addressing existing research gaps. Future studies should examine functional outcomes of moderate-load, high-volume CKC training relative to conventional training approaches in healthy adults and individuals requiring rehabilitation across the lifespan. In addition, further investigation of internal glenohumeral compression and shear force magnitudes during comparable CKC and OKC exercises may help clarify which exercise modalities are most advantageous for long-term shoulder joint health.

## 6. Conclusions

Conventional occupational demands, strength training, and rehabilitation practices for the upper extremity, often characterized by heavy loads and OKC movements, may represent an evolutionary mismatch relative to the CKC demands under which the primate shoulder evolved. Although heavy-load training can improve strength and function when applied progressively with appropriate technique and thoughtful programming, this review integrates comparative primate morphology, evolutionary considerations on the human shoulder, and functional biomechanics to propose an alternative framework for upper extremity training. This framework emphasizes high-volume, CKC exercise performed at low to moderate loads and faster movement speeds, aligning training parameters with the structural and physiological characteristics of the human shoulder. By attending to joint morphology, articular cartilage integrity, the force–velocity relationship, and evolutionary influences on muscle fiber composition, this framework offers an evolutionary-informed perspective that may help guide future research and the development of training and rehabilitation strategies aimed at promoting long-term shoulder function and joint health.

## Figures and Tables

**Table 1:** Shoulder complex morphological features in terrestrial quadrupedal primates.

1	Lateral orientation of scapula on a narrow thorax [13,18]
2	Glenoid faces ventrally [19]
3	Glenoid is pear-shaped with a superior projection [14,20]
4	Joint architecture makes flexion and extension easier than abduction and adduction [14]
5	Abduction functional center located at superior aspect of glenoid [21]
6	Supraspinatus and deltoid stabilize the glenohumeral joint during gait [19]
7	Knuckle walkers: Glenoid is positioned between a cranial and lateral orientation
8	Knuckle walkers: Adapted rotator cuff insertion pattern on humeral tubercles improve stability and limit shear. The pattern appears triangular from a superior view and is caused by lateral placement of the infraspinatus muscle [22,23]

Additional shoulder complex morphological features associated with terrestrial quadrupedal primates not detailed in the main text. Bracketed numbers indicate supporting references.

**Table 2:** Shoulder complex morphological features in suspensory-brachiating primates.

1	Longer clavicle accommodates frontal plane scapular position [30]
2	Larger supraspinatus to compensate for loss of leverage [16,17]
3	More reliance on humeral adductors for glenohumeral joint stability [31]
4	Less robust scapula, superior facing glenoid allows for ease of overhead use [19,32]
5	Abduction functional center located at superior aspect of glenoid [21]

Additional shoulder complex morphological features associated with suspensory-brachiating primates not detailed in the main text. Bracketed numbers indicate supporting references.

## Data Availability

No new data were created or analyzed in this study. Data sharing is not applicable to this article.

## Appendix A

Appendix A presents a theoretical estimation of internal glenohumeral joint pressures during a 200 lb bench press and examines their potential relationship to reported thresholds of maximal articular cartilage pressure tolerance.

A recent study demonstrated the increasing shoulder joint compression forces with different bench press grip widths [37]. The study only used a 35 lb. barbell for resistance. It showed that when moving from a narrow grip to a medium grip bench press the glenohumeral compression forces increased @ 10%. When the grip was widened to a 2.0 biacromial width, the compression forces increased 30%. With a wide grip the compressive load within the glenohumeral joint was measured at approximately 1575 N. As an example, a more realistic scenario could assume a 200 lb experienced weightlifter can press his body weight, which is 5.7 times the amount lifted in the aforementioned study. As the loads increase, it is safe to assume the pressures within the joint will increase as well. In this example the lifter would create approximately 9000 N of compression force in the glenohumeral joint if the forces only increased linearly. The thickness of articular cartilage provides evidence for the load-bearing capacity of joints. Articular cartilage is a connective tissue that covers the surfaces of synovial joints and is responsive and adaptive to mechanical load [91]. Articular cartilage has a specialized role in force dissipation and therefore, is found to be thicker in weightbearing joints. The knee joint, for example, exhibits total articular cartilage thickness of approximately 4 mm in young males [92]. Whereas, the glenohumeral joint has total articular cartilage thickness approximately 2 mm [93]. A study showed that articular cartilage cell death can occur with pressures of 2300 psi or at 16 MPa, if the force is applied very quickly and over an exceedingly small area [94]. It has been determined that the contact area of an adult glenohumeral joint is 4 to 5 cm squared [95]. If 9000 N of force was applied to the shoulder through the established contact area, then the pressures in the joint would approach 17 MPa. The force is applied over a greater time during the wide-grip bench press exercise, so these pressures are not equal in their potential to damage the articular cartilage, but it warrants consideration. This may be the reason old world terrestrially based monkeys had flattened humeral heads with increased joint contact area with the glenoid to help decrease some of the pressure within the shoulder joint. The increased surface area spread the force over a greater area.
